# The lactate-to-albumin ratio as a potential biomarker for acute kidney injury risk in critically ill patients with heart failure: a retrospective study with real-world external validation

**DOI:** 10.3389/fnut.2026.1874267

**Published:** 2026-07-31

**Authors:** Gang Luo, WeiChao Li, Wang Xu

**Affiliations:** 1 Department of Cardiology, Ganzhou Hospital-Nanfang Hospital, Southern Medical University (Ganzhou People's Hospital), Ganzhou, China; 2 Department of Cardiology, The Affiliated Ganzhou Hospital, Jiangxi Medical College, Nanchang University, Ganzhou, China; 3 Department of Anesthesiology, Affliated Qingyuan Hospital (Qingyuan People's Hospital), Guangzhou Medical University, QingYuan, Guangdong Province, China

**Keywords:** acute kidney injury, heart failure, in-hospital mortality, lactate-to-albumin ratio, real-world cohort

## Abstract

**Background:**

The lactate-to-albumin ratio (LAR) is a composite biomarker that integrates measures of nutritional status, systemic inflammation, and oxidative stress in critically ill patients. Although LAR has been validated as a predictor of acute kidney injury (AKI) in sepsis populations, its ability to identify AKI risk in the setting of heart failure has not been comprehensively evaluated.

**Methods:**

This investigation utilized the eICU Collaborative Research Database (eICU) as the internal derivation cohort, with an independent clinical cohort employed for external validation. Analytical methods included multivariable logistic and Cox proportional hazards regression, restricted cubic spline (RCS) modeling, and Kaplan–Meier survival curve analysis to examine the relationship between LAR levels and AKI as well as other adverse outcomes in patients with heart failure. The stability of the results was assessed *via* prespecified subgroup analyses, interaction testing, and multiple sensitivity analyses.

**Results:**

The final study population consisted of 5,288 patients with heart failure [median (interquartile range) age 71.0 years (61.0, 80.0); 54.3% male]. The overall AKI incidence in this critically ill heart failure cohort was 23.1%. After multivariable adjustment, elevated LAR levels remained independently associated with increased AKI risk in both the primary and external validation cohorts. Patients in the highest LAR quartile (Q4) had an AKI incidence of 28.8%, compared with 18.4% in the lowest quartile (Q1), with an adjusted OR of 1.77 (95% CI 1.45–2.16; *p* < 0.001). Subgroup analyses identified statistically significant interactions for body mass index (obese vs. non-obese), chronic kidney disease (present vs. absent), and SOFA score (≥6 vs. <6), with no other significant interactions observed. RCS analysis demonstrated a non-linear dose-response relationship between LAR and AKI, with AKI risk increasing in parallel with rising LAR levels (*p* for overall association < 0.001, *p* for non-linearity = 0.014). Kaplan–Meier cumulative risk analysis confirmed significant differences in AKI incidence across LAR quartiles (log-rank *p* < 0.001). In secondary outcome analyses, higher LAR was not associated with CRRT utilization or new-onset atrial fibrillation (NOAF), but showed an inconsistent association with increased in-hospital mortality between the two cohorts.

**Conclusions:**

This cohort study identifies elevated LAR as an independent risk factor for AKI in patients with heart failure. Further prospective validation is needed to establish its clinical value for risk stratification in this population.

## Introduction

Despite the emergence of several novel pharmacotherapies for heart failure (HF) in recent years, patient morbidity and mortality remain unacceptably high (1). Acute kidney injury (AKI) is a frequent complication, especially among HF patients requiring intensive care unit (ICU) admission (2). The heart and kidneys share intricate bidirectional relationships, with severe dysregulation leading to cardiorenal syndrome—a condition encompassing acute or chronic cardiac and renal disorders characterized by reciprocal deterioration (3, 4). Given the strong, consistent correlation between AKI and increased mortality in HF, identifying ICU patients with HF who are at elevated risk of AKI is essential for improving clinical outcomes.

For the management of AKI in heart failure, current practice guidelines recommend diuretics and continuous dialysis as foundational treatments for established AKI (5). However, emerging high-quality evidence suggests these interventions do not meaningfully improve clinical outcomes, emphasizing the growing priority of AKI prevention in this population (6). Traditional AKI diagnostic markers (serum creatinine, blood urea nitrogen, and urine output) have substantial limitations for preventive strategies, as abnormalities in these parameters typically only become detectable after AKI has already developed. These late markers provide minimal clinical value for pre-emptive intervention and do not mediate mortality reduction. While a range of novel AKI biomarkers [including Cystatin C (7), urinary microalbumin (mALB) (8), iron-binding proteins (9), α_1_ or β_2_-microglobulin (10), Retinol-binding protein, and others] have been evaluated (11), most research has focused on AKI pathophysiological pathways including hemodynamic compromise, microcirculatory impairment, inflammatory activation, and metabolic reprogramming. The pathophysiology of heart failure-associated AKI has not yet established whether nutritional status modifications are linked to AKI risk, and the role of early nutritional status in AKI onset and progression remains unclear. Given that prior studies have confirmed associations between nutritional status changes and AKI in acute pancreatitis, sepsis, and post-cardiac surgery populations, exploring this relationship in heart failure patients represents a well-justified area of investigation.

Serum albumin, the primary plasma protein, is a key biomarker for assessing nutritional status and systemic inflammation (12, 13). It plays a critical role in maintaining fluid balance and osmotic pressure between intravascular and extravascular compartments, as well as transporting hormones, medications, and trace elements (14). Low serum albumin levels are associated with functional decline, incident disease, and higher all-cause mortality across various patient groups (15, 16). Lactate, produced during anaerobic metabolism, increases significantly during tissue hypoxia or hypoperfusion, serving as a widely used indicator of disease severity and prognosis in critically ill patients with trauma, infection, or sepsis (17). However, using albumin or lactate alone has limited predictive power in complex clinical settings. The lactate-to-albumin ratio (LAR), a combined biomarker introduced recently, has shown better performance in predicting adverse outcomes than either marker alone in conditions such as acute pancreatitis (18), acute respiratory distress syndrome (19), sepsis (20), and stroke (21).

Nevertheless, investigations into the predictive utility of the LAR for AKI following HF remain scarce. Consequently, this study aims to conduct a thorough assessment of the independent prognostic significance of LAR in HF patients, a population characterized by unique underlying pathophysiological processes.

## Methods

### Data sources and research designs

To ensure clarity and standardization, this study utilized data obtained from the publicly accessible PhysioNet database. Data access was managed *via* PostgreSQL, with pertinent variables extracted through SQL queries from the associated GitHub repository. The primary cohort also incororated de-identified clinical data from the eICU Collaborative Research Database (v2.0), which documents 139,367 critically ill patients and 200,859 ICU stays across 208 U.S. healthcare facilities during 2014–2015 (22). As the eICU database is covered by HIPAA's safe harbor provisions, it was exempt from additional ethical review. Given the anonymized characteristics of the dataset, ethical review boards at the affiliated medical institution and the overseeing Institute provided waivers for the necessity of obtaining informed consent.

We performed an external validation study utilizing the Clinical Data Warehouse incorporated with the Electronic Medical Record System at Qingyuan People's Hospital (QYPH). This resource is a continuously maintained and periodically refreshed database that holds de-identified information gathered from standard clinical practice at QYPH, a prominent tertiary academic healthcare facility located in Qingyuan, Guangdong Province, China. No connections were made with any outside data repositories. The research encompassed all critically ill patients aged 18 years or older diagnosed with heart failure who underwent sequential medical management from January 2019 through July 2024. The ethics review board at QYPH granted approval for the study and exempted the need for obtaining written informed consent. Extract external validation cohort data as of April 30, 2025, to support analytical objectives.

### Data acquisition and definition

Predictor variables abstracted for the retrospective cohort analysis included patient demographic characteristics, pre-existing chronic comorbid conditions, prescribed long-term medication therapies, and admission laboratory test results. Pre-existing comorbidities were ascertained from hospital discharge records using previously validated International Classification of Diseases, Tenth Revision (ICD-10) coding protocols. For laboratory testing, we extracted and analyzed the initial laboratory panel results collected immediately upon the patient's admission to the hospital. AKI was defined using Kidney Disease Improving Global Outcomes (KDIGO) diagnostic criteria, as aligned with recommendations from the 28th Acute Disease Quality Initiative (ADQI) Workgroup consensus statement (23), and events were detected within 1 week following ICU admission. *The primary study outcome* was incident AKI. AKI staging was applied in strict adherence to KDIGO guidelines, with AKI defined as either a ≥50% increase in serum creatinine (SCr) relative to baseline within 7 days, an absolute SCr elevation of ≥0.3 mg/dL within 48 h, and/or a urine output of < 0.5 mL/kg/h sustained for 6–12 consecutive h. Baseline creatinine values were defined as the SCr measurement obtained at the time of the patient's formal heart failure diagnosis. Due to the limited availability of structured hourly urine output data in the local hospital cohort, AKI classification for this subpopulation was determined solely using SCr-based diagnostic criteria. Early AKI is defined as AKI diagnosed within the first 48 h after admission to the ICU (24). *Secondary outcomes* encompassed the requirement for continuous renal replacement therapy (CRRT), in-hospital mortality risk, as well as the incidence of new-onset atrial fibrillation (NOAF).

### Statistical analysis

Sample size was determined primarily based on the volume of eligible patient data available in the eICU database, with no formal statistical power analysis conducted for the secondary endpoints. For the single-center validation cohort, sample size calculation was based on an AKI rate of approximately 23.1%, as reported in the eICU heart failure cohort. The study aimed to recruit a total of 1,000 patients with heart failure, with a projected AKI event count of 231 cases. Given a target C-statistic of 0.8 for the predictive model and 21 candidate predictor variables selected a priori, a minimum sample size of 844 patients was required to ensure robust model development. This calculation assumed a maximum acceptable difference of 0.05 in the apparent adjusted R^2^ and a 0.05 margin of error for estimation of the model intercept (25).

Continuous variables are presented as mean ± standard deviation when normally distributed, or as median with interquartile range when the normality assumption was violated. Categorical variables are reported as absolute counts and relative percentages. For between-group comparisons, the Pearson chi-square test was used for categorical variables, the Mann-Whitney U test for continuous variables with non-normal distributions, and the independent samples *t*-test for continuous variables conforming to a normal distribution. Both unadjusted and multivariable logistic regression models were utilized to examine the linear association between serum LAR levels and incident AKI in patients admitted with heart failure. Three hierarchical models were specified for this analysis: Model 1 was the unadjusted bivariate model, including only LAR as the independent variable. Model 2 adjusted for established demographic confounders, including biological sex, chronological age, and racial category. Model 3 included all covariates from Model 2 plus additional clinical characteristics, treatment variables, and laboratory biomarkers. These additional covariates comprised initial vital signs measured within 1 h of ICU admission (heart rate, mean arterial pressure, respiratory rate, peripheral oxygen saturation, and body temperature), ICU classification (cardiovascular ICU vs. non-cardiovascular ICU), SOFA score, acute physiology and chronic health evaluation (APACHE) score, APS III, administration of life support interventions (vasopressor therapy and invasive mechanical ventilation), clinical diagnosis of sepsis at admission, pre-specified medication use (insulin, non-steroidal anti-inflammatory drugs, diuretics, beta-blockers, angiotensin receptor blockers/angiotensin-converting enzyme inhibitors, calcium channel blockers, aspirin, warfarin, heparin, clopidogrel, and statins), and pre-existing comorbidities (hypertension, prior myocardial infarction, peripheral vascular disease, chronic neurological disease, COPD, diabetes mellitus, hyperthyroidism, active cancer, chronic kidney disease, and prior atrial fibrillation), as well as routine admission laboratory parameters (white blood cell count, red blood cell count, platelet count, hemoglobin, serum sodium, serum creatinine, blood urea nitrogen, serum potassium, serum calcium, serum chloride, serum glucose, serum magnesium, troponin I, and B-type natriuretic peptide).

The cumulative incidence of AKI across LAR quartiles was plotted using Kaplan–Meier survival curves, with between-group differences assessed *via* the log-rank test. To further characterize the potential non-linear relationship between LAR levels and AKI risk, we employed logistic regression modeling with restricted cubic splines. Smooth curve fitting using a penalized spline approach with four knots was also performed to better visualize the dose-response relationship between LAR concentrations and AKI risk. We additionally conducted both unadjusted and multivariable Cox proportional hazards regression analyses to investigate the linear association between LAR levels and mortality in heart failure patients with AKI. All statistical analyses were performed using R version 4.1.1 (42) and IBM SPSS Statistics version 29.0 (IBM Corp, Armonk, NY). A two-tailed *p*-value < 0.05 was considered statistically significant for all comparisons.

## Results

### Study population and baseline characteristics

**Figure 1:**
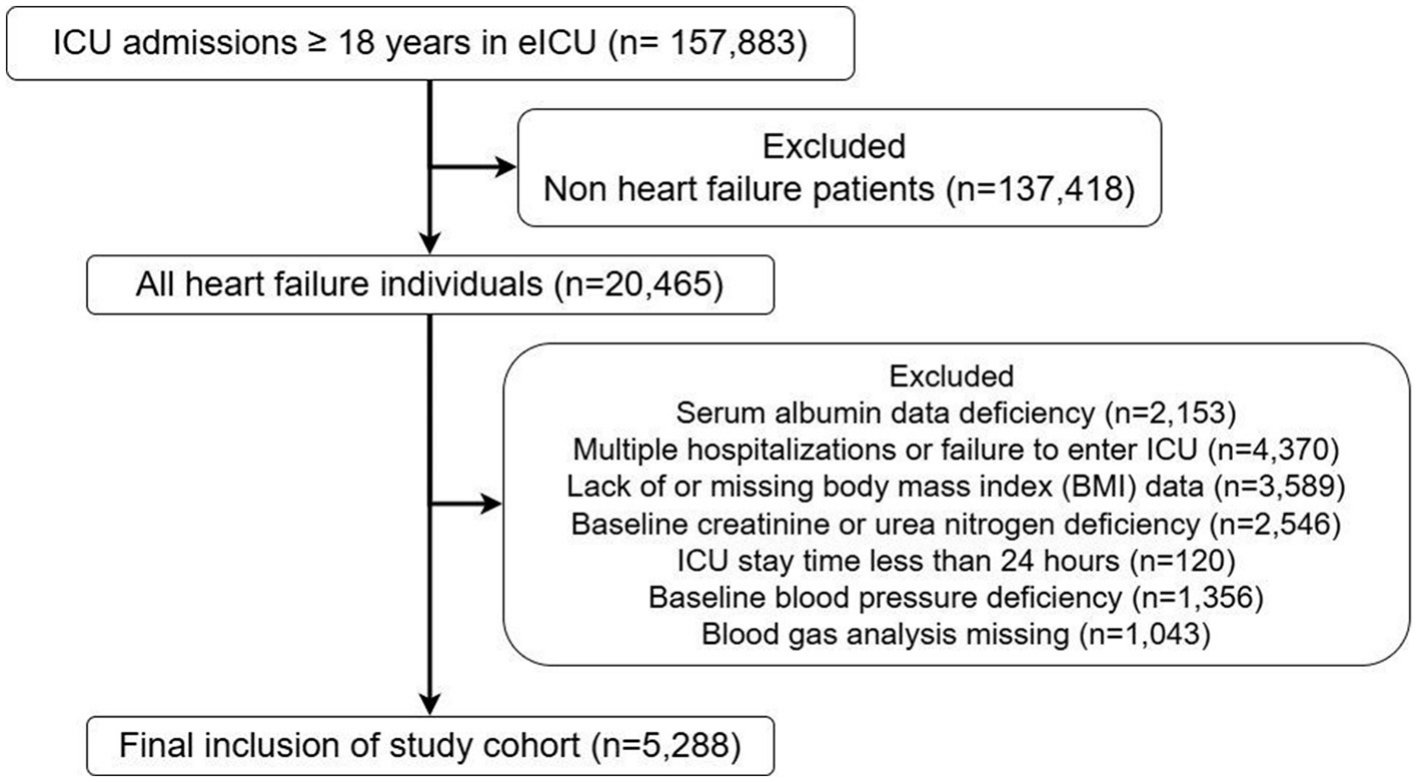
Diagram depicting the progression of participants through the study cohort.

Out of 157,883 records, 152,595 were excluded based on predefined inclusion criteria, as illustrated in Figure 1. The final cohort comprised 5,288 eligible eICU patients, with a median (IQR) age of 71.00 [61.00, 80.00] years. Among these, 2,873 (54.3%) were male, with racial/ethnic distribution as follows: 792 (14.9%) BLACK, 4,013 (75.8%) WHITE, and 483 (9.1%) categorized as OTHER/UNKNOWN. LAR levels were stratified into quartiles: Q1 (< 0.38), Q2 (0.38 to < 0.6), Q3 (0.6 to < 1.04), and Q4 (≥1.04). Relative to the Q1 group, the Q4 group exhibited an older median (IQR) age [71.00 (62.00, 81.00) years vs. 69.00 (60.00, 78.00) years] and higher median (IQR) severity scores, including APACHE [75.00 (71.00, 100.00) vs. 70.00 (55.00, 75.00)], SOFA [6.00 (5.00, 9.00) vs. 6.00 (3.00, 7.00)], and APS III [61.00 (55.00, 85.00) vs. 55.00 (40.50, 61.00)]. Additionally, the Q4 group demonstrated a higher prevalence of sepsis (49.4 vs. 30.7%) and AKI (28.8 vs. 18.4%), as detailed in Table 1 and Supplementary Table 1.

**Table 1:** Baseline characteristics in the eICU cohort categorized by lactate-to-albumin ratio quartiles.

	(Median[IQR])	LAR Quartiles				
	Overall	Q1	Q2	Q3	Q4	*p*
Covariate	*n* = 5,288	*n* = 1,295	*n* = 1,370	*n* = 1,350	*n* = 1,273	
Age, years	71.00 [61.00, 80.00]	69.00 [60.00, 78.00]	71.00 [61.00, 80.00]	72.00 [62.00, 81.00]	71.00 [62.00, 81.00]	< 0.001
Gender						
Female	2,415 (45.7)	656 (50.7)	612 (44.7)	579 (42.9)	568 (44.6)	< 0.001
Male	2,873 (54.3)	639 (49.3)	758 (55.3)	771 (57.1)	705 (55.4)	
BMI, kg/m^2^	28.90 [24.30, 35.10]	30.50 [25.00, 37.65]	29.40 [24.52, 35.90]	28.20 [23.70, 34.10]	28.00 [24.00, 33.30]	< 0.001
Heart rate, beats/min	90.00 [76.00, 101.00]	85.00 [73.00, 95.00]	89.00 [76.00, 100.00]	90.00 [78.00, 103.00]	90.00 [80.00, 106.00]	< 0.001
Mean artery pressure, mm Hg	124.00 [104.00, 139.00]	124.00 [106.00, 143.00]	124.00 [106.00, 141.00]	122.00 [103.00, 138.00]	119.00 [100.00, 134.00]	< 0.001
Respiratory rate, breaths/min	21.00 [17.00, 24.00]	20.00 [16.00, 23.00]	21.00 [17.00, 24.00]	21.00 [17.00, 25.00]	21.00 [18.00, 25.00]	< 0.001
SpO_2_ level, %	96.00 [96.00, 99.00]	97.00 [96.00, 99.00]	96.00 [96.00, 99.00]	97.00 [96.00, 99.00]	96.00 [96.00, 99.00]	0.386
Temperature, °C	36.67 [36.30, 37.10]	36.67 [36.30, 37.00]	36.70 [36.40, 37.10]	36.70 [36.40, 37.10]	36.60 [36.20, 37.10]	< 0.001
APACHE_score	75.00 [60.00, 82.00]	70.00 [55.00, 75.00]	75.00 [58.00, 77.75]	75.00 [62.00, 83.00]	75.00 [71.00, 100.00]	< 0.001
APS III_score	61.00 [46.00, 67.00]	55.00 [40.50, 61.00]	60.00 [44.00, 62.00]	61.00 [47.00, 67.00]	61.00 [55.00, 85.00]	< 0.001
SOFA	6.00 [4.00, 8.00]	6.00 [3.00, 7.00]	6.00 [4.00, 7.00]	6.00 [4.00, 7.00]	6.00 [5.00, 9.00]	< 0.001
Comorbidity						
Cancer	236 (4.5)	49 (3.8)	51 (3.7)	74 (5.5)	62 (4.9)	0.074
CKD	1,726 (32.6)	465 (35.9)	432 (31.5)	441 (32.7)	388 (30.5)	0.021
COPD	1,679 (31.8)	459 (35.4)	452 (33.0)	436 (32.3)	332 (26.1)	< 0.001
Diabetes	1,305 (24.7)	349 (26.9)	336 (24.5)	313 (23.2)	307 (24.1)	0.142
Prior AF	1,109 (21.0)	259 (20.0)	297 (21.7)	295 (21.9)	258 (20.3)	0.539
Hypertension	3,474 (65.7)	902 (69.7)	911 (66.5)	864 (64.0)	797 (62.6)	0.001
Hyperthyroidism	35 (0.7)	12 (0.9)	11 (0.8)	7 (0.5)	5 (0.4)	0.306
MI	1,058 (20.0)	237 (18.3)	288 (21.0)	307 (22.7)	226 (17.8)	0.004
Peripheral vascular disease	530 (10.0)	142 (11.0)	135 (9.9)	123 (9.1)	130 (10.2)	0.455
Chronic neurologic disease	694 (13.1)	176 (13.6)	166 (12.1)	173 (12.8)	179 (14.1)	0.464
Medications						
NSAIDS	1,251 (23.7)	328 (25.3)	327 (23.9)	345 (25.6)	251 (19.7)	0.001
Insulin	2,139 (40.5)	511 (39.5)	533 (38.9)	577 (42.7)	518 (40.7)	0.183
Betablocker	1,460 (27.6)	419 (32.4)	394 (28.8)	364 (27.0)	283 (22.2)	< 0.001
ARB/ACEI	670 (12.7)	219 (16.9)	184 (13.4)	170 (12.6)	97 (7.6)	< 0.001
CCB	363 (6.9)	137 (10.6)	99 (7.2)	68 (5.0)	59 (4.6)	< 0.001
Aspirin	1,867 (35.3)	472 (36.4)	490 (35.8)	504 (37.3)	401 (31.5)	0.01
Warfarin	445 (8.4)	137 (10.6)	123 (9.0)	115 (8.5)	70 (5.5)	< 0.001
Heparin	2,299 (43.5)	619 (47.8)	581 (42.4)	592 (43.9)	507 (39.8)	0.001
Clopidogrel	531 (10.0)	137 (10.6)	155 (11.3)	134 (9.9)	105 (8.2)	0.06
Diuretic	2,052 (38.8)	519 (40.1)	539 (39.3)	553 (41.0)	441 (34.6)	0.005
Statins	995 (18.8)	291 (22.5)	275 (20.1)	245 (18.1)	184 (14.5)	< 0.001
Laboratory tests						
Red blood cell count,/μl	3.56 [3.07, 4.03]	3.46 [2.99, 3.85]	3.59 [3.10, 4.07]	3.59 [3.11, 4.10]	3.53 [3.04, 4.06]	< 0.001
White blood cell count,/μl	12.02 [8.40, 15.95]	10.00 [7.40, 13.49]	11.70 [8.31, 14.88]	12.68 [8.73, 17.25]	13.80 [9.90, 19.40]	< 0.001
Platelet count, × 10^3^/μl	186.42 [134.50, 235.00]	190.00 [143.00, 235.00]	187.25 [136.00, 237.00]	189.00 [133.12, 237.00]	180.00 [121.00, 228.00]	0.001
Hemoglobin level, g/dL	10.55 [9.05, 12.10]	10.25 [8.88, 11.66]	10.70 [9.15, 12.30]	10.75 [9.30, 12.25]	10.50 [9.00, 12.16]	< 0.001
Calcium level, mg/dL	8.37 [7.90, 8.80]	8.50 [8.10, 8.90]	8.45 [8.00, 8.90]	8.35 [7.90, 8.80]	8.17 [7.60, 8.67]	< 0.001
Magnesium level, mEq/L	1.94 [1.70, 2.10]	1.94 [1.80, 2.20]	1.94 [1.70, 2.10]	1.90 [1.70, 2.10]	1.94 [1.70, 2.10]	0.001
Potassium level, mEq/L	4.25 [3.85, 4.75]	4.30 [3.90, 4.75]	4.20 [3.85, 4.70]	4.20 [3.80, 4.70]	4.30 [3.87, 4.90]	< 0.001
Sodium level, mEq/L	137.67 [134.60, 140.67]	138.00 [135.00, 140.67]	137.50 [134.67, 140.50]	137.50 [134.50, 140.50]	137.67 [134.00, 141.00]	0.304
Chloride level, mEq/L	102.50 [98.00, 107.00]	102.26 [98.00, 106.50]	102.25 [98.00, 106.00]	102.50 [98.00, 107.00]	103.00 [98.00, 107.33]	0.054
Ureanitrogen level, mg/dL	34.00 [22.00, 52.00]	33.00 [22.00, 53.00]	32.00 [21.00, 50.00]	35.00 [22.00, 53.00]	35.00 [23.00, 55.00]	0.007
Creatinine level, mg/dL	1.68 [1.10, 2.80]	1.66 [1.04, 3.00]	1.58 [1.00, 2.60]	1.60 [1.08, 2.66]	1.88 [1.23, 2.92]	< 0.001
Glucose level, mg/dL	158.00 [114.00, 175.25]	142.00 [107.00, 162.00]	154.00 [114.25, 170.00]	162.00 [121.00, 190.00]	162.00 [117.00, 191.00]	< 0.001
Troponin I, μg/L	2.26 [0.22, 2.26]	2.26 [0.15, 2.26]	2.26 [0.18, 2.26]	2.26 [0.29, 2.26]	2.26 [0.27, 2.26]	0.06
BNP, pg/ml						< 0.001
< 1,000	559 (10.6)	167 (12.9)	152 (11.1)	145 (10.7)	95 (7.5)	
1,000 to < 5,000	45,44 (85.9)	1,093 (84.4)	1,176 (85.8)	1,160 (85.9)	1,115 (87.6)	
≥5,000	185 (3.5)	35 (2.7)	42 (3.1)	45 (3.3)	63 (4.9)	
pCO_2_ level, mm Hg	44.27 [37.70, 45.60]	44.27 [42.00, 51.00]	44.27 [40.00, 47.00]	44.27 [36.80, 44.27]	42.00 [32.60, 44.27]	< 0.001
PH	7.42 [7.32, 9.33]	7.42 [7.32, 9.33]	7.43 [7.35, 9.33]	7.43 [7.34, 9.33]	7.38 [7.28, 9.33]	< 0.001
pO_2_ level, mm Hg	129.32 [80.00, 129.32]	125.00 [78.65, 129.32]	129.32 [79.00, 129.32]	129.32 [82.00, 129.32]	129.32 [81.00, 143.00]	< 0.001
Lactate level, mg/dL	1.60 [1.10, 2.60]	0.80 [0.70, 1.00]	1.30 [1.10, 1.60]	2.00 [1.70, 2.40]	4.10 [3.10, 6.10]	< 0.001
Albumin, g/dL	2.80 [2.30, 3.20]	3.00 [2.70, 3.40]	2.80 [2.40, 3.20]	2.60 [2.30, 3.00]	2.50 [2.10, 2.90]	< 0.001
Lactate/Albumin	0.59 [0.38, 1.00]	0.29 [0.23, 0.33]	0.48 [0.42, 0.53]	0.76 [0.67, 0.86]	1.63 [1.24, 2.47]	< 0.001

Continuous data are presented as mean ± standard deviation or median (interquartile range). Categorical data are shown as frequency (percentage). P values were calculated using one-way ANOVA, the Kruskal-Wallis H test, or the Chi-square test to evaluate differences in variables across LAR quartile groups.

LAR, lactate-to-albumin ratio; APS III, acute physiology score III; CKD, chronic kidney disease; CRRT, continuous renal replacement therapy; SOFA, sequential organ failure assessment; CKD, chronic kidney disease; AF, atrial fibrillation; APACHE, acute physiology and chronic health evaluation; ARB/ACEI, angiotensin II receptor blocker/angiotensin-converting enzyme inhibitor; CCB, calcium channel blocker; NSAIDS, non-steroidal anti-inflammatory drugs; BNP, B-type natriuretic peptide; COPD, chronic obstructive pulmonary disease; MI, myocardial infarction; BMI, body mass index.

### Association between LAR levels and AKI of critically Ill patients with heart failure

In the unadjusted model (Model 1), the highest LAR quartile (Q4) was positively associated with increased risk of AKI defined by serum creatinine (Scr) alone compared to the lowest quartile (Q1), with an odds ratio (OR) of 1.79 [95% confidence interval (CI), 1.49–2.15; *p* < 0.001; Table 2]. After adjusting for age, sex, and race (Model 2), the association remained significant (OR, 1.81; 95% CI, 1.51–2.18; *p* < 0.001). Further adjustment for a comprehensive set of confounders—including heart rate, mean arterial pressure, respiratory rate, oxygen saturation (SpO_2_), body temperature, SOFA score, APACHE score, APS III score, mechanical ventilation, vasopressor use, comorbidities, treatments, and laboratory results (Model 3)—yielded an adjusted OR of 1.77 (95% CI, 1.45–2.16; *p* < 0.001). When treated as a continuous variable, LAR continues to show a strong association with AKI (OR 1.09, 95% CI 1.03–1.15; *p* = 0.003).

**Table 2:** Analysis of primary outcomes categorized according to LAR quartiles.

	OR (95% CI) *p*				
	LAR Quartiles				
	Q1 *n* = 1,295	Q2 *n* = 1,370	Q3 *n* = 1,350	Q4 *n* = 1,273	*p*
AKI only by Scr					
Incident, (%)	239 (18.4)	301 (21.8)	301 (22.0)	390 (28.8)	< 0.001
Continuous variable	1.09 (1.03 ~ 1.15) *p* = 0.003				
Model 1	Ref	1.24 (1.03 ~ 1.50) *p* = 0.027	1.25 (1.03 ~ 1.51) *p* = 0.021	1.79 (1.49 ~ 2.15) *p* < 0.001	
Model 2	Ref	1.24 (1.03 ~ 1.50) *p* = 0.024	1.26 (1.04 ~ 1.53) *p* = 0.017	1.81 (1.51 ~ 2.18) *p* < 0.001	
Model 3	Ref	1.29 (1.06 ~ 1.57) *p* = 0.011	1.30 (1.06 ~ 1.58) *p* = 0.011	1.77 (1.45 ~ 2.16) *p* < 0.001	
AKI only by Scr and UO					
Incident, (%)	887 (68.2)	940 (68.1)	965 (70.4)	1,070 (78.9)	< 0.001
Continuous variable	1.15 (1.07 ~ 1.24) *p* < 0.001				
Model 1	Ref	1.00 (0.85 ~ 1.17) *p* = 0.972	1.11 (0.94 ~ 1.31) *p* = 0.216	1.75 (1.47 ~ 2.08) *p* < 0.001	
Model 2	Ref	1.01 (0.85 ~ 1.18) *p* = 0.947	1.14 (0.97 ~ 1.35) *p* = 0.357	1.81 (1.52 ~ 2.16) *p* < 0.001	
Model 3	Ref	1.01 (0.86 ~ 1.20) *p* = 0.556	1.08 (0.90 ~ 1.29) *p* = 0.406	1.62 (1.34 ~ 1.97) *p* < 0.001	
Early AKI					
Incident, (%)	187 (14.4)	231 (16.7)	216 (15.8)	316 (23.3)	< 0.001
Continuous variable	1.10 (1.03 ~ 1.17) *p* < 0.001				
Model 1	Ref	1.20 (0.97 ~ 1.48) *p* = 0.092	1.11 (0.90 ~ 1.38) *p* = 0.319	1.81 (1.48 ~ 2.21) *p* < 0.001	
Model 2	Ref	1.20 (0.97 ~ 1.48) *p* = 0.091	1.12 (0.90 ~ 1.38) *p* = 0.305	1.82 (1.49 ~ 2.23) *p* < 0.001	
Model 3	Ref	1.25 (1.01 ~ 1.55) *p* = 0.043	1.14 (0.91 ~ 1.42) *p* = 0.261	1.73 (1.39 ~ 2.15) *p* < 0.001	
CRRT					
Incident, (%)	150 (11.5)	138 (10.0)	141 (10.3)	162 (11.9)	0.292
Continuous variable	1.01 (0.92 ~ 1.11) *p* = 0.803				
Local hospital cohort					
	Q1 *n* = 359	Q2 *n* = 384	Q3 *n* = 377	Q4 *n* = 375	*p*
AKI only by Scr					
Incident, (%)	52 (14.5)	56 (14.6)	65 (17.2)	106 (28.3)	< 0.001
Continuous variable	1.18 (1.05 ~ 1.32) *p* = 0.004				
Model 1	Ref	1.01 (0.67 ~ 1.52) *p* = 0.970	1.23 (0.83 ~ 1.83) *p* = 0.307	2.33 (1.61 ~ 3.37) *p* < 0.001	
Model 2	Ref	0.99 (0.65 ~ 1.49) *p* = 0.951	1.25 (0.84 ~ 1.87) *p* = 0.276	2.35 (1.62 ~ 3.42) *p* < 0.001	
Model 3	Ref	1.09 (0.62 ~ 1.95) *p* = 0.759	1.07 (0.60 ~ 1.90) *p* = 0.819	1.81 (1.03 ~ 3.18) *p* = 0.038	
CRRT					
Incident, (%)	150 (11.5)	138 (10.0)	141 (10.3)	162 (11.9)	0.292
Continuous variable	1.01 (0.92 ~ 1.11) *p* = 0.803				

Categorical data are presented as frequency (percentage). P values for outcome comparisons across LAR quartile groups were derived using chi-square tests.

All analytical models for the eICU dataset were developed using logistic regression. Model I: Unadjusted baseline model. Model II: Adjusted for demographic factors, specifically biological sex, chronological age, and racial classification. Model III: Adjusted for all variables in Model II plus a comprehensive set of clinical and laboratory variables. These included initial vital signs recorded ICU admission (heart rate, mean arterial pressure, respiratory rate, peripheral oxygen saturation, and body temperature), ICU type (cardiovascular vs. non-cardiovascular), severity-of-illness scores (SOFA, APACHE, and APS III), receipt of critical interventions (vasopressor support and invasive mechanical ventilation), a diagnosis of sepsis upon admission, use of specific medications (insulin, NSAIDs, diuretics, beta-blockers, ARBs/ACE inhibitors, calcium channel blockers, aspirin, warfarin, heparin, clopidogrel, and statins), and a history of comorbidities (hypertension, prior myocardial infarction, peripheral vascular disease, chronic neurological disease, COPD, diabetes mellitus, hyperthyroidism, active cancer, chronic kidney disease, and prior atrial fibrillation). Additionally, standard laboratory values from admission were incorporated, covering white blood cell count, red blood cell count, platelet count, hemoglobin, and serum levels of sodium, creatinine, blood urea nitrogen, potassium, calcium, chloride, glucose, magnesium, troponin I, and B-type natriuretic peptide.

All logistic regression analyses for the local institutional cohort were adjusted for the complete set of collected covariates, including demographic characteristics, preexisting medical comorbidities, medication use history, and routine admission laboratory values.

LAR, lactate-to-albumin ratio; Scr, serum creatinine; UO, urine output; CRRT, continuous renal replacement therapy; AKI, acute kidney injury.

Furthermore, we investigated the association between LAR and AKI using two alternative definitions. In Model 1, the highest quartile Q4 of LAR was positively associated with an elevated risk of AKI defined by both Scr and urine output (UO) criteria compared to the lowest Q1 (OR, 1.75; 95% CI, 1.47–2.08; *p* < 0.001; Table 2). After adjustment for age, sex, and race in Model 2, the OR was 1.81 (95% CI, 1.52–2.16; *p* < 0.001). Following full adjustment for all confounders in Model 3, the association remained statistically significant (adjusted OR, 1.62; 95% CI, 1.34–1.97; *p* < 0.001). Additionally, in Model 1, Q4 LAR was positively associated with an increased risk of early AKI compared to Q1 (OR, 1.81; 95% CI, 1.48–2.21; *p* < 0.001; Table 2). After adjusting for age, sex, and race in Model 2, the OR was 1.82 (95% CI, 1.49–2.23; *p* < 0.001). Full adjustment for all confounders in Model 3 resulted in an adjusted OR of 1.73 (95% CI, 1.39–2.15; *p* < 0.001; Table 2).

There was no statistically significant association between LAR quartile status and CRRT utilization when comparing the highest and lowest quartiles (adjusted OR 1.01, 95% CI 0.92–1.11; *p* = 0.803). When viewed as a continuous variable, LAR continues to exhibit strong independence from CRRT (Table 2).

### Restricted cubic spline (RCS) regression and cumulative probability analysis

**Figure 2:**
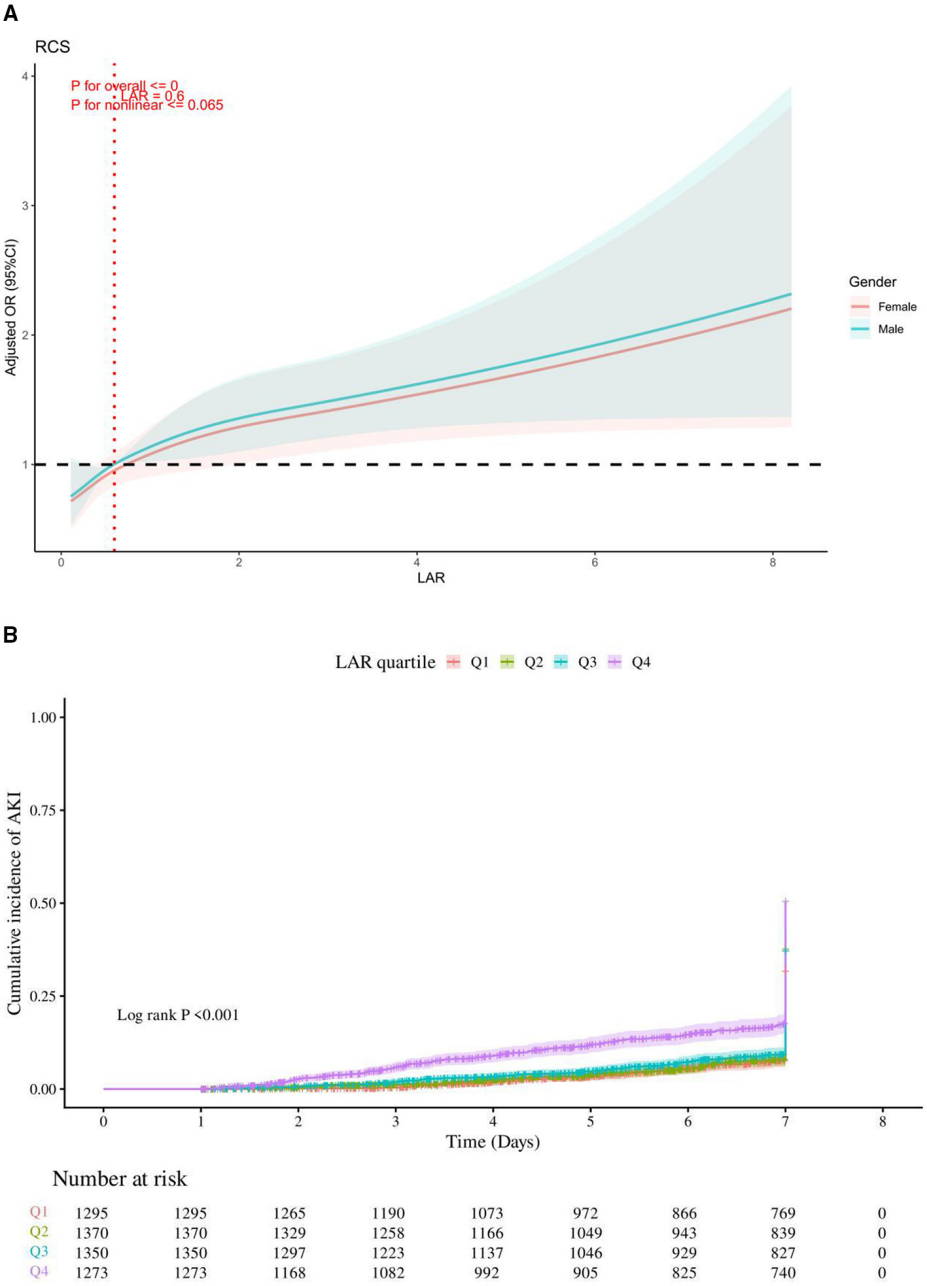
Adjusted RCS models illustrating the relationship between LAR levels and the risks of AKI **(A)** in the eICU cohort and **(B)** Kaplan-Meier curves demonstrating the association between LAR quartiles and AKI risk in the eICU cohort. LAR, lactate-to-albumin ratio; AKI, Acute kidney injury.

To assess the relationship between LAR levels and AKI, restricted cubic spline and multivariable logistic regression models were utilized. As shown in Figure 2, following adjustment for all pertinent covariates, a statistically significant association was found between LAR levels and AKI in patients with HF (*p* for overall association < 0.001, *p* for nonlinearity = 0.014). The analysis revealed an initial inflection point at a LAR level of 0.59; above this threshold, elevated LAR levels (Q4) correlated with a gradually increasing risk of AKI. Concurrently, the Kaplan-Meier cumulative probability analysis, presented in Figure 2B, reinforced these results by indicating that higher LAR levels were associated with greater cumulative AKI rates, with the cumulative risk of AKI rising in tandem with increasing LAR levels.

### Subgroup analysis

**Figure 3:**
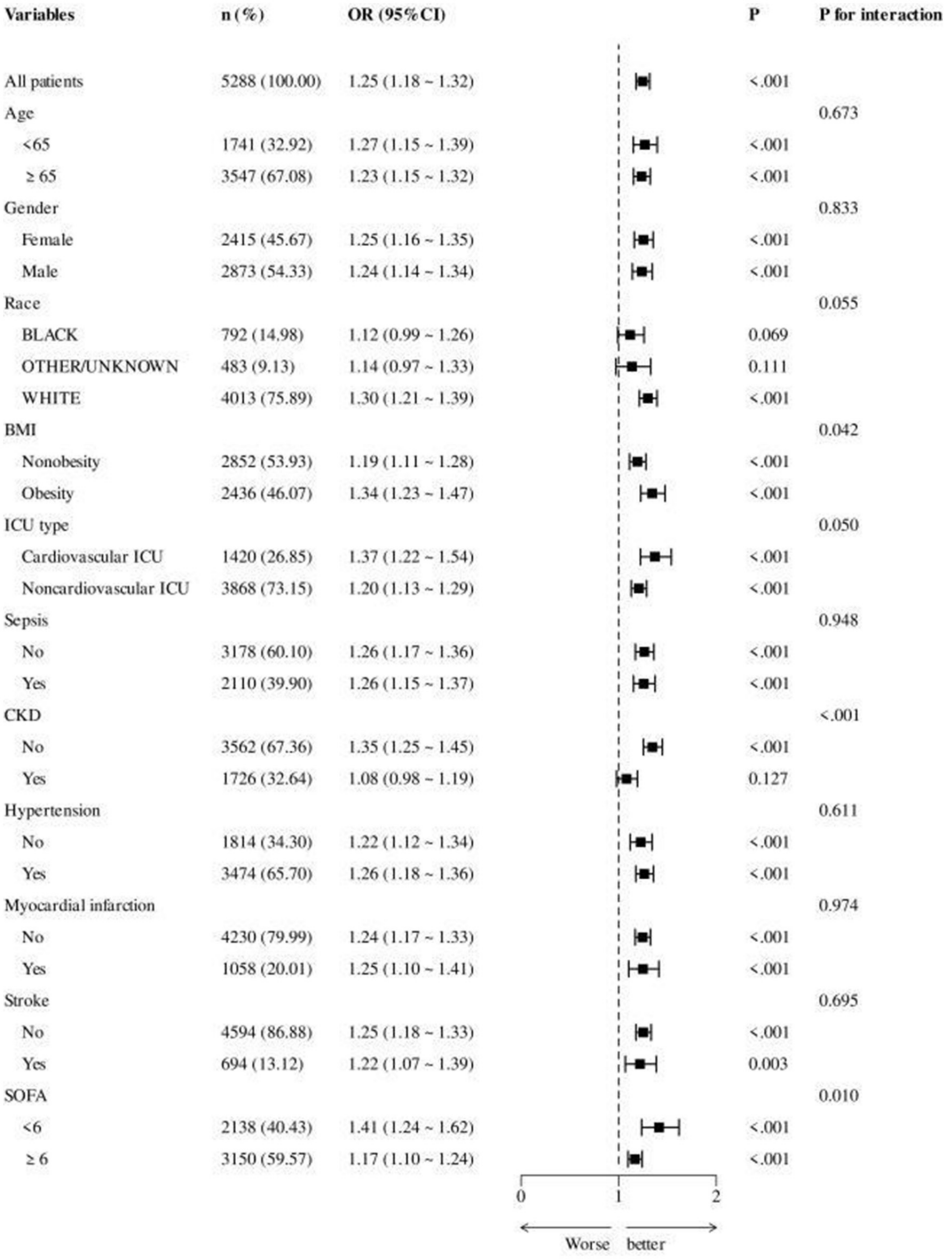
Stratified subgroup analysis investigating the association between LAR levels and the risk of AKI. Adjustments in logistic regression models included a comprehensive set of clinical and laboratory variables.

In our subgroup and interaction analyses, we examined the association between LAR levels and the risk of AKI across different patient categories. These subgroups were stratified by variables including age, race, BMI, ICU type, SOFA scores, sepsis status, CKD, myocardial infarction, hypertension, and stroke history (Figure 3). The risk of AKI did not show a statistically significant elevation in certain subgroups. For BLACK patients, the odds ratio was 1.12 (95% CI 0.99–1.26, *p* = 0.069); for patients of OTHER/UNKNOWN race, the OR was 1.14 (95% CI 0.97–1.33, *p* = 0.111); and for those with CKD, the OR was 1.08 (95% CI 0.98–1.19, *p* = 0.127). In contrast, several factors demonstrated a significant increase in AKI risk: patients younger than 65 years (OR 1.27, 95% CI 1.15–1.39, *p* < 0.001), WHITE patients (OR 1.30, 95% CI 1.21–1.39, *p* < 0.001), patients managed in a cardiovascular ICU (OR 1.37, 95% CI 1.22–1.54, *p* < 0.001), obesity (OR 1.34, 95% CI 1.23–1.47, *p* < 0.001), SOFA scores below 6 (OR 1.41, 95% CI 1.24–1.62, *p* < 0.001), and hypertension (OR 1.26, 95% CI 1.18–1.36, *p* < 0.001). Significant interaction effects were observed for BMI (*p* = 0.042), CKD (*p* < 0.001), and SOFA (*p* = 0.01), while no other interactions reached statistical significance.

### Analysis of secondary outcomes

For the in-hospital mortality endpoint (Table 3), among heart failure patients with AKI defined by SCR criteria alone, the unadjusted Model 1 showed that patients in the highest LAR quartile (Q4) had a 5.65-fold higher risk of in-hospital mortality compared with those in the lowest quartile (Q1; HR 5.65, 95% CI 4.01–7.97; *p* < 0.001; Table 3). After adjustment for demographic and baseline covariates (age, sex, ICU type, BMI, and race) in Model 2, the association remained statistically significant (HR 5.46, 95% CI 3.87–7.70; *p* < 0.001). Further adjustment for a comprehensive set of clinical confounders, including initial admission vital signs, life support interventions, severity-of-illness scores (SOFA, APACHE, APS III), comorbidities, treatments, and laboratory parameters, in Model 3 yielded an adjusted HR of 4.07 (95% CI 2.80–5.90; *p* < 0.001). When LAR was analyzed as a continuous variable, it remained strongly associated with in-hospital mortality (HR 1.25 per unit increase, 95% CI 1.18–1.32; *p* < 0.001). In supplementary subgroup analyses: Among heart failure patients with AKI defined by both SCR and urine output criteria, the highest LAR quartile was associated with a significantly higher mortality risk compared with the lowest quartile (adjusted HR 2.99, 95% CI 2.42–3.70; *p* < 0.001). Among heart failure patients with early AKI, the adjusted HR for in-hospital mortality comparing the highest to lowest LAR quartile was 4.44 (95% CI 2.81–7.03; *p* < 0.001).

**Table 3:** Analysis of the association between lactate-to-albumin ratio and mortality risk in patients with acute kidney injury or requiring continuous renal replacement therapy.

HR (95% CI) *p*					
eICU cohort	AKI only by Scr subgroup				
	LAR Quartiles				
	Q1 *n* = 305	Q2 *n* = 307	Q3 *n* = 305	Q4 *n* = 307	*p*
Incident, (%)	40 (13.1)	82 (26.7)	95 (31.1)	179 (58.3)	< 0.001
Continuous variable	1.25 (1.18 ~ 1.32) *p* < 0.001				
Model 1	Ref	2.12 (1.46 ~ 3.10) *p* < 0.001	2.34 (1.61 ~ 3.38) *p* < 0.001	5.65 (4.01 ~ 7.97) *p* < 0.001	
Model 2	Ref	2.01 (1.38 ~ 2.94) *p* < 0.001	2.20 (1.52 ~ 3.19) *p* < 0.001	5.46 (3.87 ~ 7.70) *p* < 0.001	
Model 3	Ref	1.74 (1.18 ~ 2.57) *p* < 0.001	1.93 (1.30 ~ 2.85) *p* < 0.001	4.07 (2.80 ~ 5.90) *p* < 0.001	
	AKI only by Scr and UO subgroup				
	Q1 *n* = 938	Q2 *n* = 949	Q3 *n* = 959	Q4 *n* = 960	
Incident, (%)	105 (11.2)	180 (19.0)	242 (25.2)	400 (41.7)	< 0.001
Continuous variable	1.22 (1.17 ~ 1.27) *p* < 0.001				
Model 1	Ref	1.68 (1.32 ~ 2.14) *p* < 0.001	2.28 (1.81 ~ 2.87) *p* < 0.001	3.92 (3.16 ~ 4.86) *p* < 0.001	
Model 2	Ref	1.62 (1.27 ~ 2.06) *p* < 0.001	2.13 (1.70 ~ 2.69) *p* < 0.001	3.77 (3.04 ~ 4.69) *p* < 0.001	
Model 3	Ref	1.41 (1.10 ~ 1.81) *p* < 0.001	1.77 (1.39 ~ 2.25) *p* < 0.001	2.49 (1.96 ~ 3.15) *p* < 0.001	
	Early AKI subgroup				
	Q1 *n* = 1,295	Q2 *n* = 1,370	Q3 *n* = 1,350	Q4 *n* = 1,273	
Incident, (%)	26 (11.0)	63 (26.9)	84 (35.1)	143 (60.6)	< 0.001
Continuous variable	1.23 (1.16 ~ 1.31) *p* < 0.001				
Model 1	Ref	2.51 (1.59 ~ 3.97) *p* < 0.001	3.16 (2.04 ~ 4.91) *p* < 0.001	7.00 (4.61 ~ 10.64) *p* < 0.001	
Model 2	Ref	2.35 (1.48 ~ 3.71) *p* < 0.001	3.07 (1.97 ~ 4.77) *p* < 0.001	6.66 (4.38 ~ 10.13) *p* < 0.001	
Model 3	Ref	2.44 (1.52 ~ 3.94) *p* < 0.001	2.87 (1.78 ~ 4.62) *p* < 0.001	4.44 (2.81 ~ 7.03) *p* < 0.001	
	CRRT subgroup				
	Q1 *n* = 150	Q2 *n* = 136	Q3 *n* = 139	Q4 *n* = 147	
Incident, (%)	19 (12.7)	22 (16.2)	35 (25.2)	62 (42.2)	< 0.001
Continuous variable	1.29 (1.12 ~ 1.49) *p* < 0.001				
Model 1	Ref	1.06 (0.57 ~ 1.96) *p* < 0.001	1.79 (1.02 ~ 3.14) *p* < 0.001	3.13 (1.87 ~ 5.25) *p* < 0.001	
Model 2	Ref	1.04 (0.56 ~ 1.93) *p* = 0.899	1.68 (0.95 ~ 2.97) *p* = 0.073	3.10 (1.84 ~ 5.22) *p* < 0.001	
Model 3	Ref	0.85 (0.44 ~ 1.66) *p* = 0.636	1.39 (0.73 ~ 2.64) *p* = 0.321	2.05 (1.08 ~ 3.90) *p* < 0.001	
Local hospital cohort					
AKI only by Scr subgroup *n* = 279					
Continuous variable	1.09 (0.98 ~ 1.22) *p* =0.110				
CRRT subgroup *n* = 97					
Continuous variable	1.19 (1.00 ~ 1.41) *p* = 0.057				

All analytical models for the eICU dataset were constructed using Cox proportional hazards regression, with three hierarchical adjustment levels:

Model I: Unadjusted crude model, including only the primary exposure variable.

Model II: Adjusted for basic demographic factors: sex, age, and racial identity.

Model III: Adjusted for all covariates in Model II plus a comprehensive panel of clinical and laboratory variables. These included initial vital signs obtained (heart rate, mean arterial pressure, respiratory rate, peripheral oxygen saturation, and body temperature), ICU type (cardiovascular vs. non-cardiovascular), severity of illness scores (SOFA, APACHE, and APS III), use of life support interventions (vasopressor support and invasive mechanical ventilation), admission diagnosis of sepsis, documented use of medication classes, pre-existing comorbid conditions, and routine admission laboratory parameters. Medication classes included insulin, NSAIDs, diuretics, beta-blockers, ARB/ACE inhibitors, calcium channel blockers, aspirin, warfarin, heparin, clopidogrel, and statins. Comorbidities included hypertension, prior myocardial infarction, peripheral vascular disease, chronic neurological disease, COPD, diabetes mellitus, hyperthyroidism, active cancer, chronic kidney disease, and prior atrial fibrillation. Laboratory parameters included white blood cell count, red blood cell count, platelet count, hemoglobin, serum sodium, serum creatinine, blood urea nitrogen, serum potassium, serum calcium, serum chloride, serum glucose, serum magnesium, troponin I, and B-type natriuretic peptide.

Survival analyses for the local institutional dataset were conducted using Cox proportional hazards regression models, with adjustment for all collected covariates including demographic factors, preexisting comorbidities, medication use, and routine admission laboratory test results.

LAR, lactate-to-albumin ratio; HR, hazard ratio; CI, confidence interval; CRRT, continuous renal replacement therapy; AKI, acute kidney injury.

After multivariable adjustment, the highest LAR quartile (Q4) was independently associated with an increased risk of new-onset AF compared with the lowest quartile (Q1), yielding an adjusted OR of 1.39 (95% CI 1.06–1.82; *p* = 0.016). But, restricted cubic spline and multivariable logistic regression models were not found association of LAR and NOAF (*p* for overall association = 0.47, *p* for nonlinearity = 0.346; Supplementary Table 2).

### ROC analysis

ROC analysis showed LAR's AUC for predicting AKI-only Scr was 0.565 (95% CI: 0.546–0.584), indicating statistically significant but weak discrimination. At the Youden-optimized cutoff, sensitivity was 0.282 and specificity 0.819—LAR better identifies non-events than events. Calibration: Brier score = 0.176; intercept = 0.002; slope = 1.002; Hosmer-Lemeshow test nonsignificant (χ^2^ = 8.566, df = 8, *p* = 0.380), confirming good calibration. Reclassification: continuous NRI = 0.192 (95% CI: 0.137–0.256); IDI = 0.012 (95% CI: 0.007–0.020). However, NRI was driven by downward reclassification of non-events (NRI_nonevent = 0.521), while event reclassification was negative (NRI_event = −0.328), indicating poor event risk elevation. Thus, LAR shows modest association with AKI-only Scr but limited standalone discriminatory power (Supplementary Figures 3–6).

### Validation cohort analysis

A total of 1,495 consecutive eligible patients with confirmed heart failure were included in the external validation cohort, with a mean age of 64 [52.00, 73.00] years and males accounting for 60.1% of the population. The overall AKI incidence in this clinical cohort was 18.7%. Patients were divided into four equal groups using LAR quartile thresholds, with AKI incidence highest in the highest quartile (Q4: 28.3%), followed by Q3 (17.2%), and lowest in the lowest quartile (Q1: 14.5%) (Supplementary Table 1; Supplementary Figure 1).

In the unadjusted Model 1, patients in the highest LAR Q4 had a 2.33-fold higher odds of SCR-defined AKI compared with those in the lowest Q1 (OR 2.33, 95% CI 1.61–3.37; *p* < 0.001; Table 2). After adjustment for age, sex, and race in Model 2, the magnitude of the association remained nearly unchanged and statistically significant (OR 2.35, 95% CI 1.62–3.42; *p* < 0.001). In the fully adjusted Model 3, which included additional confounders such as initial admission vital signs, life support interventions, comorbid conditions, treatment exposures, and laboratory parameters, the association remained significant with an adjusted OR of 1.81 (95% CI 1.03–3.18; *p* = 0.038). When modeled as a continuous variable, each 1-unit increase in LAR was associated with an 18% higher odds of AKI (OR 1.18, 95% CI 1.05–1.32; *p* = 0.004).

Restricted cubic spline models and multivariable logistic regression were used to characterize the relationship between LAR levels and AKI risk. As illustrated in Supplementary Figure 2A, after adjusting for all clinically relevant confounders, LAR concentrations remained significantly associated with AKI development in patients with heart failure (*p* for overall association < 0.001, *p* for non-linearity = 0.492). Kaplan-Meier cumulative risk analysis, presented in Supplementary Figure 2B, further supported these results: higher LAR levels were linked to increased cumulative AKI incidence, with progressive elevations in AKI risk detected across increasing LAR quartiles (log-rank *p* < 0.001).

In secondary outcome analyses, which differed from primary analysis results, LAR was not significantly associated with all-cause mortality risk in heart failure patients complicated by AKI (adjusted HR 1.09, 95% CI 0.98–1.22; *p* = 0.110; Table 3). LAR also showed no statistically significant association with mortality risk in heart failure patients requiring CRRT (adjusted HR 1.19, 95% CI 1.00–1.41; *p* = 0.057) and NOAF (adjusted OR 0.98, 95% CI 0.81–1.20; p = 0.856) (Supplementary Table 2).

## Discussion

Across two independent cohorts of critically ill patients with heart failure, LAR elevation emerged as an independent risk factor for AKI, with the association holding true under different AKI definitions. Subgroup and interaction analyses demonstrated that the prognostic value of LAR was influenced by race, BMI, CKD, and SOFA score. Our results were further validated through sensitivity analyses. For secondary clinical endpoints, LAR showed an uncertain association with in-hospital mortality and was not predictive of CRRT requirement or new-onset atrial fibrillation in either cohort.

AKI in the critically ill heart failure patients developed in 22.8% of patients in the eICU cohort and 18.7% local hospital cohort, similar to the rates of 23.0% (26), 25.0% (27), and 23% of previous studies (28). The data presented indicate that this study possesses a reasonable degree of representativeness within its defined scope and population.

As a metabolic end product produced during cellular hypoxia and anaerobic respiration, lactate is widely employed as a biomarker to detect inadequate tissue perfusion and the onset of metabolic acidosis (29). In various clinical environments, elevated serum lactate concentrations have been consistently linked to adverse clinical outcomes in critically ill patients. Nevertheless, interpreting lactate levels clinically is complicated by numerous physiological and pathological variables, which reduces its reliability as an independent prognostic indicator. Previous research in animal models and human subjects has demonstrated that substantial rises in lactate levels can occur in the context of acute kidney injury; potential underlying mechanisms may include disruptions in lactate metabolism, such as enhanced production, reduced clearance, and impairment of humoral or cellular immune responses (30). Albumin, the predominant plasma protein in circulation, serves a critical function in regulating colloid osmotic pressure and acts as a key indicator of nutritional status and systemic inflammatory response (31). Reduced albumin levels are commonly observed in critically ill individuals, often resulting from accelerated protein catabolism, diminished hepatic production, or significant leakage of proteins from the vascular compartment. As a result, hypoalbuminemia is well-recognized as a marker of increased disease severity and elevated mortality. A recent systematic review additionally highlighted that hypoproteinemia is linked to a heightened risk of AKI development (32). The LAR is an integrated metric that simultaneously captures information on metabolic stress, nutritional status, and systemic inflammatory burden. Variations in this composite ratio may offer a more precise reflection of the global pathophysiological state in critically ill patients, potentially delivering superior predictive value compared to either lactate or albumin measured independently. For these reasons, this combined biomarker is attracting increasing interest from both frontline clinicians and clinical investigators.

In prespecified subgroup analyses, we found no significant association between LAR levels and AKI development among BLACK patients; this null finding may be explained by genetic variations that influence AKI risk in this population. It is widely recognized that BLACK individuals in the US have higher rates of chronic kidney disease compared to populations without recent African ancestry. Two well-documented pathogenic coding variants in the APOL1 gene, G1 and G2, are responsible for a substantial proportion of the increased risk of progressive non-diabetic kidney disease observed in BLACK Americans relative to European American populations (33, 34). Furthermore, our analyses indicated that acute exacerbations of chronic kidney disease, which predominantly present as acute kidney injury, may not be associated with LAR concentrations. This result suggests that worsening nutritional status is unlikely to contribute directly to the pathogenesis of these acute CKD exacerbations.

We also found a statistically significant interaction between obesity and non-obesity, as well as between low and high SOFA scores. Obesity is an established risk factor for AKI, but BMI does not adequately account for risk related to sarcopenia (loss of skeletal muscle mass and function) (35), which can cause overweight and obese individuals to qualify for a malnutrition diagnosis despite elevated body weight. These patients face combined risks from both malnutrition and obesity-related pathophysiological processes, both of which can increase kidney injury burden. This dual risk profile may explain why LAR shows a consistent association with AKI across both obese and non-obese patient groups. This dual risk phenomenon is also observed in patients with both low and high SOFA scores. A high SOFA score typically reflects more frequent antibiotic administration to control active infection, while a low SOFA score is associated with less frequent use of antibiotics for infectious indications. LAR also partially mirrors systemic inflammatory activity, and effective infection control in critically ill patients may attenuate the excess AKI risk linked to malnutrition.

While the LAR has been established as a predictor of mortality across various disease states, its specific association with AKI has been less extensively explored. A notable investigation by Yiming Hua and colleagues, involving 5,222 patients with sepsis, demonstrated that an elevated LAR serves as an independent predictor for the development of AKI within this cohort (36). Further supporting this link, additional research has highlighted the prognostic utility of the LAR, as well as the NEWS-Lactate score, in forecasting sepsis-associated AKI (37). The relevance of LAR extends to other clinical contexts; for instance, it has been shown to correlate with both the occurrence and prognosis of AKI following cardiac surgery (38). In the setting of percutaneous coronary intervention, Ji-Lang Zeng reported that LAR was significantly and independently associated with the incidence of contrast-associated AKI (39). Beyond predicting AKI onset, LAR also holds prognostic value for outcomes post-AKI. A study by Xiaoyun Shi and colleagues, which included 7,100 patients diagnosed with AKI, identified an elevated LAR as an independent predictor of mortality in this population (40). Similarly, research by Jianfei Liu and colleagues examined the association between the lactate/albumin ratio and prognosis in critically ill patients with AKI who were undergoing continuous renal replacement therapy (41). Although our study population comprised patients with heart failure, the association between LAR levels and mortality risk in critically ill heart failure patients with concomitant acute kidney injury showed inconsistent results across the two study cohorts. More randomized controlled trials will be required to confirm and characterize this relationship in future investigations. For remaining secondary outcomes, we detected no significant association between LAR and either CRRT receipt or NOAF, suggesting that systemic inflammation or malnutrition is not associated with these endpoints. Existing clinical guidelines for NOAF management do not recognize malnutrition or inflammation as established risk factors for the development of this arrhythmia. Similarly, standard clinical indications for CRRT include hyperkalemia, severe acidosis, rising serum creatinine, and severe fluid retention, but do not incorporate nutritional status or inflammatory burden as triggering criteria.

## Limitations

Several important limitations of this work should be noted. First, despite efforts to adjust for a comprehensive set of potential confounding variables, the retrospective observational design cannot fully account for unmeasured residual confounding. Second, nutritional status was assessed only at ICU admission, which prevents analysis of how changes in nutritional status over the course of hospitalization may be associated with the study endpoints. Third, we did not adjust for inflammatory markers or other biological pathways that may mediate the relationship between nutritional status and clinical outcomes. Fourth, detailed anthropometric data including body composition parameters (e.g., body fat percentage, lean body mass) and pre-admission physical activity levels were not available, limiting our ability to elucidate the specific mechanisms driving the observed association between nutritional status and outcomes. Finally, variability in patient selection criteria across heart failure studies focused on nutritional status may contribute to differences in reported outcome risks across study cohorts.

## Conclusion

This cohort analysis demonstrates that elevated LAR levels are consistently associated with increased AKI risk across two distinct ICU cohorts of patients with heart failure, supporting the generalizability of our study findings. These results suggest that optimal nutritional status and controlled systemic inflammation may provide a protective benefit against AKI development in critically ill patients with heart failure. Prospective randomized controlled trials are warranted to further validate these findings and test interventional approaches.

## Data Availability

The original contributions presented in the study are included in the article/Supplementary material, further inquiries can be directed to the corresponding authors.
