# Supplementary material for: The lactate-to-albumin ratio as a potential biomarker for acute kidney injury risk in critically ill patients with heart failure: a retrospective study with real-world external validation
Source: Front Nutr. 2026 Jul 31;13:1874267. doi: 10.3389/fnut.2026.1874267 (PMC13472818; doi:10.3389/fnut.2026.1874267)
Supplement: Supplementary file 1 [file Table_1.docx]

**Supplementary**

**Supplementary Table 1.**

Baseline characteristics in the local hospital cohort categorized by lactate-to-albumin ratio quartiles.

**Supplementary Table 2.**

Analysis of the Association Between Lactate-to-Albumin Ratio and New-onset Atrial Fibrillation.

**Supplementary Figure 1.**

Diagram depicting the progression of participants through the local hospital cohort.

**Supplementary Figure 2.**

Adjusted RCS models and cumulative risks or Kaplan-Meier curves depict the relationship between LAR levels and the risks of outcomes in the local hospital cohort.

**Supplementary Figure 3.** Receiver operating characteristic curve for the prediction of AKI only Scr using LAR.

**Supplementary Figure 4.** Calibration plot of the LAR-based model for predicting AKI only Scr.

**Supplementary Figure 5.** Decision curve analysis of the LAR-based model for predicting AKI only Scr.

**Supplementary Figure 6**. Distribution of predicted probabilities according to AKI only Scr.

**Supplementary Table 1.**

Baseline characteristics in the local hospital cohort categorized by lactate-to-albumin ratio quartiles.

|  | **(Median [IQR])** |  |  |  |  |  |
| --- | --- | --- | --- | --- | --- | --- |
|  | **Overall** | **Q1** | **Q2** | **Q3** | **Q4** | **p** |
| **Covariate** | **n=1495** | **n=359** | **n=384** | **n=377** | **n=375** |  |
| Age, years | 64.00 [52.00, 73.00] | 65.00 [54.00, 74.00] | 62.50 [52.00, 72.00] | 63.00 [51.00, 72.00] | 66.00 [54.00, 74.00] | 0.043 |
| Gender |  |  |  |  |  | 0.566 |
| Female | 597 (39.9) | 133 (37.0) | 154 (40.1) | 152 (40.3) | 158 (42.1) |  |
| Male | 898 (60.1) | 226 (63.0) | 230 (59.9) | 225 (59.7) | 217 (57.9) |  |
| ICU type |  |  |  |  |  | 0.414 |
| Cardiovascular ICU | 549 (36.7) | 145 (40.4) | 135 (35.2) | 133 (35.3) | 136 (36.3) |  |
| Noncardiovascular ICU | 946 (63.3) | 214 (59.6) | 249 (64.8) | 244 (64.7) | 239 (63.7) |  |
| BMI, kg/m^2^ | 25.38 [25.38, 25.38] | 25.38 [25.38, 25.38] | 25.38 [25.38, 25.38] | 25.38 [25.38, 25.38] | 25.38 [25.38, 25.38] | 0.889 |
| Heart rate, beats/min | 96.00 [87.00, 98.00] | 96.00 [81.00, 96.00] | 96.00 [84.00, 100.00] | 96.00 [93.00, 99.00] | 96.00 [91.00, 100.50] | <0.001 |
| Mean artery pressure,mm Hg | 82.00 [76.00, 83.00] | 82.00 [77.00, 87.00] | 82.00 [76.00, 86.00] | 82.00 [77.00, 82.00] | 82.00 [75.00, 82.00] | 0.014 |
| Respiratory rate, breaths/min | 21.00 [18.00, 21.00] | 21.00 [17.00, 21.00] | 21.00 [18.00, 22.00] | 21.00 [19.00, 21.00] | 21.00 [21.00, 23.00] | <0.001 |
| SpO_2_ level, % | 97.00 [97.00, 99.00] | 97.00 [97.00, 100.00] | 97.00 [97.00, 100.00] | 97.00 [97.00, 99.00] | 97.00 [97.00, 99.00] | 0.022 |
| Temperature, °C | 36.56 [36.56, 36.80] | 36.56 [36.40, 36.90] | 36.56 [36.55, 36.90] | 36.56 [36.56, 36.80] | 36.56 [36.50, 36.70] | 0.01 |
| CRRT | 97 ( 6.5) | 15 ( 4.2) | 21 ( 5.5) | 29 ( 7.7) | 32 ( 8.5) | 0.063 |
| Ventilation | 726 (48.6) | 149 (41.5) | 172 (44.8) | 180 (47.7) | 225 (60.0) | <0.001 |
| Vasopressor | 900 (60.2) | 190 (52.9) | 210 (54.7) | 236 (62.6) | 264 (70.4) | <0.001 |
| Cirrhosis | 55 ( 3.7) | 14 ( 3.9) | 9 ( 2.3) | 20 ( 5.3) | 12 ( 3.2) | 0.169 |
| CKD | 47 ( 3.1) | 11 ( 3.1) | 10 ( 2.6) | 13 ( 3.4) | 13 ( 3.5) | 0.892 |
| Diabetes | 81 ( 5.4) | 22 ( 6.1) | 21 ( 5.5) | 19 ( 5.0) | 19 ( 5.1) | 0.908 |
| Stroke | 54 ( 3.6) | 14 ( 3.9) | 15 ( 3.9) | 8 ( 2.1) | 17 ( 4.5) | 0.321 |
| Chroniclungdisease | 77 ( 5.2) | 17 ( 4.7) | 26 ( 6.8) | 9 ( 2.4) | 25 ( 6.7) | 0.02 |
| Sepsis | 150 (10.0) | 26 ( 7.2) | 32 ( 8.3) | 44 (11.7) | 48 (12.8) | 0.035 |
| ARB/ACEI | 219 (14.6) | 56 (15.6) | 63 (16.4) | 59 (15.6) | 41 (10.9) | 0.13 |
| Betablocker | 573 (38.3) | 147 (40.9) | 144 (37.5) | 142 (37.7) | 140 (37.3) | 0.71 |
| CCB | 316 (21.1) | 75 (20.9) | 81 (21.1) | 87 (23.1) | 73 (19.5) | 0.684 |
| Diuretic | 789 (52.8) | 204 (56.8) | 208 (54.2) | 190 (50.4) | 187 (49.9) | 0.188 |
| Statins | 565 (37.8) | 154 (42.9) | 169 (44.0) | 127 (33.7) | 115 (30.7) | <0.001 |
| White blood cell count, /μl | 13.70 [8.90, 19.20] | 12.40 [8.30, 17.40] | 13.45 [9.07, 18.90] | 13.90 [9.10, 19.50] | 14.80 [8.70, 21.35] | 0.02 |
| Creatinine level, mg/dL | 1.13 [0.85, 1.80] | 1.01 [0.81, 1.52] | 1.06 [0.80, 1.61] | 1.12 [0.86, 1.68] | 1.37 [1.02, 2.35] | <0.001 |
| Ureanitrogen level, mg/dL | 21.00 [14.00, 36.00] | 21.00 [14.00, 37.00] | 20.00 [14.00, 33.00] | 20.00 [14.00, 32.00] | 25.00 [17.00, 42.50] | <0.001 |
| Albumin, g/dL | 3.00 [2.50, 3.40] | 3.10 [2.70, 3.50] | 3.10 [2.60, 3.50] | 2.90 [2.50, 3.40] | 2.70 [2.20, 3.10] | <0.001 |
| Lactate level, mg/dL | 3.80 [2.90, 5.70] | 2.70 [2.30, 3.00] | 3.30 [2.90, 3.80] | 4.30 [3.70, 5.20] | 7.90 [6.35, 10.40] | <0.001 |
| Lactate/Albumin | 1.25 [0.97, 1.93] | 0.88 [0.84, 0.91] | 1.08 [1.00, 1.15] | 1.47 [1.35, 1.65] | 2.74 [2.26, 3.84] | <0.001 |
| Potassium level, mEq/L | 4.10 [3.60, 4.50] | 4.10 [3.70, 4.55] | 4.00 [3.60, 4.50] | 4.00 [3.60, 4.40] | 4.10 [3.60, 4.75] | 0.116 |
| Sodium level, mEq/L | 139.00 [136.00, 142.00] | 139.00 [136.00, 142.00] | 139.00 [135.00, 141.00] | 139.00 [135.00, 142.00] | 140.00 [135.00, 143.00] | 0.091 |

**Supplementary Table 2.**

Analysis of the Association Between Lactate-to-Albumin Ratio and New-onset Atrial Fibrillation.

|  | **OR (95% CI) *p*** | |  |  |  |
| --- | --- | --- | --- | --- | --- |
|  | **Q1** | **Q2** | **Q3** | **Q4** | ***p*** |
| **eICU** |  |  |  |  |  |
| **Incident, (%)** | 137 (10.5) | 184 (13.3) | 195 (14.2) | 225 (16.6) | <0.001 |
| **Continuous variable** | 1.08 (1.00 ~ 1.16)  *p*=0.064 | |  |  |  |
| **Categorical variable** | Ref | 1.18 (0.91 ~ 1.52)  *p*=0.027 | 1.14 (0.88 ~ 1.48)  *p*=0.327 | 1.39 (1.06 ~ 1.82)  *p*=0.016 |  |
| **Local hospital cohort** |  |  |  |  |  |
| **Incident, (%)** | 14 ( 3.9) | 18 ( 4.7) | 17 ( 4.5) | 19 ( 5.1) | 0.897 |
| **Continuous variable** | 0.98 (0.81 ~ 1.20)  *p*=0.856 | |  |  |  |
| **Categorical variable** | Ref | 1.21 (0.59 ~ 2.47)  *p*=0.598 | 1.16 (0.56 ~ 2.40)  *p*=0.681 | 1.32 (0.65 ~ 2.66)  *p*=0.447 |  |

**Supplementary Figure 1.**

Diagram depicting the progression of participants through the local hospital cohort.

| **Supplementary Figure 2.**  Adjusted RCS models and cumulative risks or Kaplan-Meier curves depict the relationship between LAR levels and the risks of outcomes in the local hospital cohort. | |
| --- | --- |
| AKI | |
| (A) | (B) |
|  |  |

**Supplementary Figure 3. Receiver operating characteristic curve for the prediction of AKI only Scr using LAR.**

The receiver operating characteristic curve illustrates the discriminative performance of the lactate-to-albumin ratio (LAR) for predicting AKI only Scr. The area under the curve (AUC), equivalent to the C-statistic, was 0.565 (95% CI: 0.546-0.584), indicating weak discriminative ability. The dashed diagonal line represents no discrimination. The optimal threshold based on the Youden index corresponded to a sensitivity of 0.282 and a specificity of 0.819.

**Supplementary Figure 4. Calibration plot of the LAR-based model for predicting AKI only Scr.**

The calibration plot compares the predicted probabilities from the LAR-based logistic model with the observed event rates across risk groups. The calibration intercept was 0.002 and the calibration slope was 1.002, with a Brier score of 0.176. The Hosmer-Lemeshow goodness-of-fit test showed no evidence of poor fit (χ²=8.566, df=8, p=0.380), suggesting acceptable apparent calibration.

**Supplementary Figure 5. Decision curve analysis of the LAR-based model for predicting AKI only Scr.**

Decision curve analysis was performed to evaluate the clinical net benefit of the LAR-based prediction model across a range of threshold probabilities. The LAR model was compared with default strategies of treating all patients and treating no patients. The model provides potential clinical utility only in threshold ranges where its net benefit exceeds both reference strategies, and the modest AUC suggests that its standalone clinical usefulness is limited.

**Supplementary Figure 6**. Distribution of predicted probabilities according to AKI only Scr.

The distribution of predicted probabilities is shown separately for patients with and without AnyAKIonlyScr. The substantial overlap between the two distributions indicates limited separation between event and non-event groups, consistent with the weak discriminative performance observed in the ROC analysis. Continuous NRI and IDI analyses suggested modest incremental improvement over the intercept-only model, mainly driven by improved downward reclassification among non-events.
